# Origins, characteristics and destination of nursing students in South West England

**DOI:** 10.1186/s12912-023-01210-2

**Published:** 2023-03-18

**Authors:** K. Hambridge, S. Banerjee, L. Winfield, J. Gripton

**Affiliations:** 1 University of Plymouth, 7 Portland Villas, Drake Circus, PL4 8AA Plymouth, UK; 2 University of Plymouth, Plymouth, UK; 3 Cornwall Partnership NHS Foundation Trust, Bodmin, UK

**Keywords:** Supply, Demand, Modelling, Workforce, Nursing, Nursing students

## Abstract

**Background:**

Worldwide there are concerns about the supply of nurses into health systems. Understanding and balancing the supply of and demand for healthcare professionals is crucial to efficient healthcare delivery, yet there is relatively little research that examines in detail where nursing students come from and where they go after qualification.

**Objectives:**

To investigate the demographic characteristics of applicants to nursing and midwifery programmes in England, those that are enrolled, attrition during study, and their career intentions on graduation.

**Methods:**

A descriptive case study was conducted in south west England drawing on a complementary set of analyses of routinely collected application and enrolment data from 2017–2020. These were augmented by derivation of student deprivation indices and a follow-up study of nursing and midwifery students qualifying between May 2020 and April 2021.

**Results:**

The percentage of males applying for nursing doubled and the mean age of all enrolled students (except midwifery) increased during the study period. The mean level of deprivation of applicants increased from the 51^st^ to the 55^th^ centile indicating widening of participation. Most applying and enrolled students originated from the same region as the nursing school and remained working there on qualification. Successively more males than females were lost from the system at each stage from application to qualification. Qualifying students most common job choice was within acute Trusts, with Medical, District nursing and surgical being the most common choices. The most important factors regarding job choice were location, the characteristics of the Trust, having been there as a student, and family considerations.

**Conclusions:**

The data provide useful information on the nursing educational pipeline. The data discussed here raise questions that would benefit from further regional and national empirical research.

## Background

Worldwide there are concerns about the supply of nurses into health services [1]. Such worries about the capacity of the nursing profession in England to meet clinical demand have been part of the nursing workforce discourse at least since *The Nurses Act* established the first professional register in the UK in 1919 [2]. In England, there are more than 40,000 reported nursing vacancies in hospital and community health services [3, 4]. These shortages in the nursing workforce have been identified as the most crucial NHS staff shortfall to be addressed and they include important geographical variation [4]. The reasons for this are multifactorial, including deficiencies in workforce planning, cuts in bursaries to students for training limiting supply, and pay restraint leading to nurses leaving the profession [5]. Additional supply side constraints include demographic changes in numbers of young people leaving school [6] and restrictive immigration policies exacerbated by Brexit [7]. In 2017, applicants dropped by 18–33% [6, 8] with fewer more mature applicants [9]. In 2018, there was a further 9–13% decline on 2017 levels [6, 8]. With recent changes in funding, reinstating a maintenance grant, there are indications that student recruitment increased in 2020 [10]. An additional factor affecting the supply of registered nurses is attrition among pre-registration nursing students and the retention of newly qualified nurses [11, 12]. The attrition rate was reported to be 19% in the early 2000s [11], with some suggestion of this increasing in the last decade to 24–33% in samples of universities [6, 13].

Understanding and balancing the supply of and demand for healthcare professionals is crucial to efficient healthcare delivery [14], yet there is relatively little research that examines in detail where nursing students come from, where they go after qualification, and the determinants of enrolment in nursing studies and career decision-making, before and after studies. We therefore carried out a programme of analyses to start to address this evidence gap and generate data that would help develop a dynamic model to improve future nursing workforce planning and modelling in the South West of England.

The objectives of the project were, using data from a single university in South West England (the University of Plymouth), to:

- investigate the demographic characteristics, including patterns of deprivation, of applicants to nursing and midwifery programmes
- explore the career intentions of graduating nursing and midwifery students
- calculate the attrition rates from nursing and midwifery programmes
- determine the level of transfers to nursing and midwifery programmes

## Methods

### Setting

This study was conducted at the University of Plymouth in the South West of England where nursing degree courses in adult nursing, child nursing, mental health nursing, and midwifery are delivered on three sites in Devon and Cornwall (Plymouth, Exeter, and Truro).

### Design

The form of the research is a descriptive case study. This drew on a complementary set of analyses of routinely collected application and enrolment data from 2017–2020, augmented with derivation of student deprivation indices from home postcode data. A follow-up study of nursing and midwifery students qualifying between May 2020 and April 2021 was added. The use of different samples aided triangulation and thus the validity of the data. A descriptive in-depth case study methodology was adopted in order to build the foundations of understanding of nursing student flows. This design ‘allows in-depth, multi-faceted explorations of complex issues in their real-life settings’ [15] and for the incorporation of multiple data sources to generate a single coherent narrative, while acknowledging the potential for limitations in generalisability from the specific area studied. The programme was divided into four interlocking work packages (WP):

- WP1 covered applications, and students enrolled to pre-registration nursing and midwifery programmes between 2017–2020, focussing on demographic data and deprivation. This was data available on a database of applications and enrolments of students held by the university. We used home postcode data to generate Indices of Multiple Deprivation 2019 [16] to assign level of deprivation. The system links postcodes to Lower-layer Output Areas and generates a deprivation score between one and 32,844, with one the most deprived area and 32,844 the least deprived. Postcodes from Wales, Northern Island, Scotland, Channel Islands and outside the England could not be processed using this system.
- WP2 examined these data for those who enrolled onto nursing programmes from 2018/19 to 2020/21. This was data available on a database of applications and enrolments of students held by the university.
- WP3 consisted of an online exit survey of final year students completing nursing and midwifery programmes during the summer of 2020. The survey was designed in order to capture their origins on application, their career choices and reasons for this choice.
- WP4 examined the attrition rates from nursing and midwifery programmes between 2011–2019 and the numbers and type of transfers into Year 2 of those programmes from 2016–2019. This was data available on a database of applications and enrolments of students held by the university.

Data were managed using Excel spreadsheets and extracted into SPSS Statistics 25 for analysis [17]. All data were anonymised after necessary information was extracted and stored securely on a password-protected hard-drive. We computed the mean proportions of sex, mean age, and deprivation score in each of the three samples. We compared applicants with enrolled students and enrolled students with those graduating. Descriptive statistics including quartiles were generated. We used t-tests to identify differences in proportions with *p* < 0.05 regarded as significant.

## Results

### WP1 and WP2 – Demographics, origin, and deprivation of applicants and enrolled students 2017–2020

The data covered 5,017 applications over the four-year period. The details are presented in Table 1.

**Table 1:** WP1 Applications to nursing courses 2017–2020

	**Adult**	**Child**	**Midwifery**	**Mental Health**	**Total**
**Total applications (n)**					
2017	406	145	189	92	832
2018	739	243	309	213	1505
2019	765	292	282	221	1560
2020	653	132	159	176	1120
Total	2563	812	939	702	5017
**Sex female n / (%)**					
2017	371 (91.4)	141 (97.2)	187 (98.9)	81 (88.1)	780 (93.8)
2018	670 (90.7)	232 (95.5)	308 (99.7)	179 (84.0)	1389 (92.4)
2019	679 (88.8)	281 (96.2)	281 (99.7)	173 (78.3)	1414 (90.6)
2020	551 (84.4)	126 (95.5)	159 (100)	129 (73.3)	965 (86.2)
Total	2271 (88.6)	780 (96.1)	935 (99.6)	562 (80.1)	4548 (90.7)
**Mean age / (age range)**					
2017	25.9 (19–52)	22.5 (19–50)	25.2 (19–52)	23.0 (19–49)	24.8 (19–52)
2018	26.6 (18–63)	21.9 (18–45)	24.8 (18–52)	26.2 (18–60)	25.4 (18–63
2019	25.2 (17–56)	21.9 (17–50)	24.8 (18–52)	27.7 (17–62)	24.7 (17–62)
2020	27.5 (15–56)	23.8 (16–56)	23.7 (17–51)	29.1 (17–56)	26.8 (15–56)
Total	26.3 (15–63)	22.3 (16–56)	24.4 (17–52)	27.0 (17–62)	25.4 (15–63)
**Mean deprivation score**^**a**^** / (n)**					
2017	14,703 (364)	18,431 (125)	18,110 (174)	15,466 (83)	16,208 (745)
2018	15,243 (672)	18,012 (212)	16,600 (280)	17,229 (193)	16,236 (1355)
2019	15,964 (665)	15,630 (243)	17,016 (256)	14,481 (188)	15,898 (1350)
2020	14,650 (476)	16,157 (99)	16,248 (144)	12,941 (130)	14,836 (847)
Total	15,243 (2177)	16,966 (676)	16,674 (854)	15,175 (591)	15,849 (4297)
**Region of origin n / (%)**					
South West	1,956 (85.5)	542 (71.7)	741 (80.1)	498 (78.9)	3,737 (81.2)
South East	115 (5.0)	75 (9.9)	68 (7.4)	42 (6.7)	300 (6.5)
Wales	54 (2.4)	51 (6.7)	31 (3.4)	29 (4.6)	165 (3.6)
West Midlands	43 (1.9)	28 (3.7)	21 (2.3)	20 (3.2)	112 (2.4)
London	25 (1.1)	16 (2.1)	21 (2.3)	8 (1.3)	70 (1.5)
Other	96 (4.2)	44 (5.8)	43 (4.6)	34 (5.4)	217 (4.7)

^a^Indices of Multiple Deprivation 2019

The number of applications increased by 13.5% over the four study years from 832 to 1120. 4,548 (90.7%) were female, with the percentage of males lowest in Midwifery (*n* = 935, 0.4%) and Child Nursing (*n* = 780, 3.9%) and highest in Mental Health (*n* = 562, 19.1%). The percentage of males applying for nursing programmes between 2017 and 2020 more than doubled overall from 6.2% to 13.8%. This was driven by applicants to Adult and Mental Health Nursing which increased from 9.6% to 15.6% and from 11.9 to 26.7% respectively. The patterns were similar in the enrolled students with 92.7% (*n* = 1,627) overall female. The increase in male applicants was not reflected in increased admissions in Adult Nursing, but it was in Mental Health Nursing. On graduation the percentage of males had dropped to 4% overall, but was higher within degree apprentices (10%) and Mental Health (12%). This can be seen in Table 2.

**Table 2:** WP2 Students enrolled onto nursing courses 2018/19–2020/21

	**Adult**	**Nursing Associate**	**Child**	**Midwifery**	**Mental Health**	**Total**
**Total enrolments**						
2018/19	376	0	58	57	63	554
2019/20	267	113	51	65	68	564
2020/21	333	85	54	84	80	636
Total	976	198	163	206	211	1754
**Sex – female n / (%)**						
2018/19	349 (92.8)	0	58 (100)	56 (98,2)	58 (92.0)	521 (94.0)
2019/20	245 (91.7)	106 (93.8)	50 (98.0)	65 (100)	54 (79.4)	520 (92.2)
2020/21	305 (91.5)	78 (91.7)	51 (94.4)	84 (100)	68 (85.0)	586 (92.1)
Total	899 (92.0)	184 (93.0)	159 (97.5)	205 (99.5)	180 (85.0)	1627 (92.7)
**Mean age / (age range)**						
2018/19	26.5 (17–54)	0	21.6 (17–44)	25.1 (17–45)	21.8 (17–42)	25.3 (17–54)
2019/20	24.8 (17–54)	32 (18–57)	21.7 (17–39)	25.9 (17–51)	27.0 (17–50)	26.3 (17–57)
2020/21	25.9 (17–56)	31 (18–57)	21.5 (17–48)	24.9 (17–43)	26.3 (17–50)	26.2 (17–57)
Total	25.9 (17–56)	31.6 (18–57)	21.6 (17–48)	25.3 (17–51)	25.2 (17–53)	26.0 (17–57)
**Mean deprivation score**^**a**^**/ (n)**						
2018/9	14,401 (258)	-	17,175 (49)	16,657 (57)	16,179 (44)	15,241 (408)
2019/20	16,277 (192)	-	15,856 (50)	17,172 (61)	13,672 (55)	16,003 (368)
2020/21	14,895 (131)	-	20,636 (13)	17,953 (25)	12,900 (27)	15,391 (196)
Total	15,132 (581)	unavailable	16,988 (112)	16,861 (143)	14,473 (156)	15,399 (1232)
**Region of origin n / (%)**						
South West	911 (93.2)	196 (98.5)	144 (92.3)	192 (93.7)	191 (91.4)	1,634 (93.2)
South East	19 (1.9)	1 (0.5)	6 (3.8)	1 (0.5)	8 (3.8)	35 (2.0)
West Midlands	10 (1.0)	0	3 (1.9)	4 (2.0)	4 (1.9)	21 (1.2)
Africa	13 (1.3)	0	2 (1.3)	1 (0.5)	0	16 (0.9)
Wales	4 (0.4)	0	1 (0.6)	3 (1.5)	1 (0.5)	9 (0.5)
Other	20 (2.0)	2 (1.0)	8 (5.1)	4 (2.0)	5 (2.4)	39 (2.2)

^a^Indices of Multiple Deprivation 2019

The number of enrolments increased by 14.8% from 554 to 636 over the four study years.

There was a wide age range with the greatest proportion of mature students in Adult, Nursing Associate and Mental Health Nursing. The trend was for an increase in the mean age of all groups (25.3 – 26.2) other than Midwifery (25.1 – 24.9) where there was a decrease over the study period.

The origin data demonstrate the regional nature of the School of Nursing and Midwifery. The large majority of applicants (3,737/4,600. 81.2%) were from the South West region with 3,298 (88.3%) of these from the three counties closest to the School (Devon, Cornwall, and Somerset). The pattern was similar in the enrolled students but there was a higher likelihood of regional residence in those enrolled than in applicants (93.2% v 81.2%, χ^2^ = 137.9, *p* < 0.001).

The mean level of deprivation of the applicants from 2017–2020 increased from the 51^st^ to the 55^th^ centile (16,208 – 14,836) where the highest deprivation centile (100^th^) corresponds to the lowest one percent of the areas of deprivation (i.e. 1 to 489). There was a variation in the mean deprivation score of applicants by field with the highest deprivation in mental health (54^th^ centile, 15,175) and adult nursing students (54^th^ centile, 15,243), and the lowest in Midwifery (49^th^ centile, 16,674) and Child nursing (48^th^ centile, 16,966). The mean deprivation score of applicants was 15,849 (52^nd^ centile), for enrolled students was 15,399 (53^rd^ centile), and for qualifying students was 16,103 (51^st^ centile).

### WP3 –Survey of graduating students 2020

A questionnaire was sent via email to 550 3rd year nursing and midwifery students as they were about to complete their programmes. The survey ran from 7th July 2020 to 20th August 2020. There were 298 responses, giving a response rate of 54.2%. The results of this are summarised in Table 3.

**Table 3:** Survey of final year students completing nursing programmes in 2020 (*n* = 298)

	**Adult** n (%)	**Degree apprenticeship** n (%)	**Child** n (%)	**Midwifery** n (%)	**Mental Health** n (%)	**Total** n (%)
**Number of respondents**	196	10	32	34	26	298
**Age on application n / (%)**						
17–19	42 (21)	0	15 (47)	9 (27)	11 (42)	77 (26)
20–29	83 (42)	3 (30)	12 (35)	13 (38)	9 (35)	120 (40)
30–39	44 (22)	4 (40)	4 (13)	9 (27)	5 (19)	66 (22)
40–49	24 (12)	2 (20)	0	3 (9)	1 (4)	30 (10)
50 +	3 (2)	1 (10)	1 (3)	0	0	5 (2)
**Sex – female** **n / (%)**	189 (96)	9 (90)	30 (94)	34 (100)	23 (88)	285 (96)
**Home county on application n / (%)**						
South West	180 (91.8)	10 (100)	29 (90.6)	30 (88.2)	23 (88.5)	272 (91.3)
Wales	3 (1.5)	0	1 (3.1)	0	1 (3.8)	5 (1.7)
South East	2 (1.0)	0	0	2 (5.8)	0	4 (1.3)
West Midlands	2 (1.0)	0	0	0	1 (3.8)	3 (1.0)
London	2 (1.0)	0	0	0	0	2 (0.7)
Other	7 (3.5)	0	2 (6.2)	2 (5.8)	1 (3.8)	12 (4.0)
**Mean deprivation score**^a^** / (n)**	15,726 (176)	15,093 (9)	16,596 (26)	16,421 (29)	18,333 (24)	16,103 (264)
**First job destination type n / (%)**						
Acute	131 (74)	6 (60)	22 (100)	22 (100)	2 (9)	183 (72)
Community/ mental health	45 (25.4)	4 (40)	0	0	18 (82)	67 (27)
Prison	0	0	0	0	2 (9)	2 (1)
Navy	1 (0.6)	0	0	0	0	1 (0)
**First job location n / (%)**						
Local^b^	166 (88.3)	10 (100)	16 (61.5)	24 (85.7)	20 (83.3)	236 (86)
Non-local	22 (11.7)	0	10 (38.5)	4 (14.3)	4 (16.7)	40 (14)

^a^Indices of Multiple Deprivation 2019

^b^Cornwall, Devon and Somerset

The sample was in line with the applicant data obtained in WP2 and WP3 with 96% of the sample female and 40% (n = 120) aged between 20–29 years. Comparing deprivation scores of those graduating with those that enrolled, there were similar scores in Adult nursing (15,726 compared with 15,243), Child nursing (16,596 v 16,996), and Midwifery (16,421 v 16,679), but those graduating in Mental Health nursing had lower deprivation scores than those enrolled (18,333 (45^th^ centile) v 15,175 (54^th^ centile)).

The most common specialty for the student’s first job was Medical (27.7%), followed by District Nursing (14.2%) and Surgical (10.2%). Acute Trusts (72.3%) which provide secondary health services, were most popular, followed by Mental Health and Community Trusts (26.5%). In terms of destination, 236 (86%) of the 253 students where destination after graduation data were available went to work locally in Devon, Cornwall, and Somerset. Most students were from the local area (253/297) and went to work in the local area (225/276, 81.5%). Only 13/238 (5.5%) local students left the area after qualifying to work, and 11/38 (28.9%) non-local students stayed to work locally. The most important factors identified by the students when choosing their first job were: location (including financial reasons) (223/298, 74.8%), the characteristics of the Trust (142/298, 47.7%), experience of being there as a student (91/298, 30.5%), and family considerations (48/298, 16.1%).

### WP4 – Attrition rates 2011–2019 and transfers in 2016–2019

The overall mean attrition rate for all fields over the time frame was 9.2% based upon the proportion of students enrolled completing their studies. The fields with the highest mean attrition rates were Adult Nursing (13.4%) and Mental Health (13.2%), and Nursing Associates (10.5%). The fields with the lowest mean attrition rates were Child (0.17%), Degree Apprenticeships (0.9%) and Midwifery (4.8%). Over 2016–2019 there were 93 students who transferred into nursing programmes from other universities, an average of 23 per year, equivalent to 5.3% of enrolments.

### Comparisons of applicants, enrolled and graduating students

The 95% confidence interval (95%CI) for the difference between the proportion of male applicants (9.3%) and male enrolled students (7.1%) was 2.2% (95%CI 3.7% to 0.8%). For the difference between enrolled (7.1%) and qualifying (5.4%) students the difference between the proportions was 1.7% (95%CI 5.4% to 0.2%). This suggests that successively more males are lost from the system at each stage from application to qualification.

## Discussion

By triangulating data from multiple datasets, we have generated a preliminary dynamic model of the flow of nursing students from application to the destination of their first clinical post. It is striking that while male applications increased in the study period, they were less likely than female applicants to be selected for enrolment, and less likely to graduate once enrolled. The data also illustrate the essentially regional nature of the students’ origin and destination, with over 90% of enrolled students from the South West England and 86% of graduates going on to work in the three counties closest to the university. This illustrates the central contribution of nursing schools to the regional health economy. The number of applications increased by 13.5% over the four study years and this was mirrored by a 14.8% rise in enrolments.

In terms of the efficiency of nursing education in delivering registered nurses, we found that fewer than 10% of those enrolled left before qualification and this was balanced in part by transfers into the courses equivalent to 5.3%. The overall mean attrition rate for all fields over the time frame was 9.2% based upon the proportion of students enrolled completing their studies. Data of this sort modelling the system has potential value in workforce planning.

### Student characteristics

Male nurses make up a small minority of the nursing population, with estimates made for England being 12% [18]. The need to address gender-based bias in nursing training has been identified [19]. It is of interest that in this study we observed a doubling of males applying for nursing programmes, but that this was not reflected in the proportion of men enrolled. This requires further study since it may be that there are barriers inherent in the nursing selection system that men find more difficult to cross than women. The observed inequality in enrolment may be an issue that is local to the system or may be part of the wider set of system-wide gender issues in nursing training. It is also possible that the males applying were less likely to meet specific gender-fair criteria. Further research using geographically wider, and therefore more generalisable, data that also seeks to understand reasons for rejection would be useful and the results could help build local and national response to address inequity if needed.

Data within Tables 1 and 2 showed that there is a bimodal distribution of applicants and enrolments to nursing with a group of students who apply within a year or two of completing school and a group of more mature applicants. The age profiles of the applicants and enrolments here show that Child and Midwifery students were more likely to be straight out of school while more mature applicants chose Adult, Mental health, and Nursing Associate courses with 40.4% of enrolled and 40.3% of graduating students aged between 20–29 on application. The benefits of including older students include their being more likely to complete [20, 21], and remain in the profession [9], as well as their bringing different skills and experiences compared with younger peers to their learning sets [22].

Over the study period there was a trend for applications to come from more deprived backgrounds, but this was a relatively small change overall, from the 51st to the 55th centile (16,208 – 14,836). The most competitive courses drew from areas of lower deprivation than others, with applicants to Midwifery (49th centile, 16,674) and Child nursing (48th centile, 16,966) having mean deprivation ratings that were lower than the national average while those applying to mental health (54th centile, 15,175) and adult nursing (54th centile, 15,243), had mean deprivation above the national average, but again the absolute difference was relatively modest. Nursing students need to reflect the populations they serve, and the move observed towards recruitment from areas of relatively higher deprivation makes an important contribution to social mobility and widening participation [9].

### Attrition

There will always be some who enrol on nursing programmes, for whom nursing may emerge as not the right path for them at that stage. This may be on academic or personal grounds. The loss of students during training is a concern for universities and workforce planners alike [18] due to financial and workforce reasons. In this cohort the mean attrition rate for all programmes was 9.2%. As others have reported [11], there was considerable heterogeneity in attrition rates between courses with very low rates in the most competitive and therefore selective courses (Child nursing (0.2%) and Midwifery (4.8%)) and the highest rates in larger, less selective courses (Adult Nursing (13.4%) and Mental Health (13.2%), and Nursing Associates (10.5%)). Interestingly the new Degree Apprenticeship courses had a low attrition rate (0.9%). The attrition rates observed here compare favourably with The Health Foundation’s [6] analysis of data from 58 of 74 UK universities offering nursing degrees which showed an average attrition rate of 24% for nursing and 21% for midwifery. In generating a dynamic model of attrition and its effect on the supply of registered nurses it is also useful to consider transfers between universities as well as those that leave. In our study between 2016–2019 an average of 23 students per year transferred in from other universities, making our net attrition equivalent to 3.9% of enrolments.

### Student mobility and destination

Regarding the mobility of qualifying students, the data showed that most of our qualifying students were local and stayed in the local area. In a study of 162 Australian nursing students [23], there was a strong correlation between where the student originated and where they wanted to work, hence the importance of ‘growing your own.’ Our data are also in line with reports that students who lived locally were more likely to successfully progress [20].

The most common specialty for the student’s first job was Medical, followed by District Nursing and Surgical. Understandably because of the relative sizes of the courses offered, acute hospitals were found to be most popular, followed by mental health and community services. The literature on student nurse career decisions in the UK is limited but suggests that students on qualification have a preference for fast paced acute environments [24]. Similarly, a study of 456 final year students showed that the most popular career choice for newly qualified nurses was to work in acute areas such as emergency and critical care, while primary care and care of the elderly were less popular [25]. Similar findings have been reported in Australia [26, 27].

Regarding reasons for choosing the first job, the most important factors reported were: location, the characteristics of the Trust, having been there as a student, and family considerations. This fits with findings that mature students and students with personal caring responsibilities are more likely to stay in their existing location [9]. It is clear that where the Trust is located in relation to the student’s home, and the experience that they may have had when working there as a student, are key determinants in deciding whether to work there once qualified. Understandably, student nurses state preferences for where they have been treated well [28] and that they would not work in areas where they perceived there to be insufficient support [23]. There are clear messages for recruitment of newly qualified nursing staff. The more students they take and the better the experience they provide for them, the greater the likelihood is that those students will choose to work with them once qualified. Those who live locally may be the most productive investments.

### Limitations and strengths

The main limitation of this study is that it is of a single nursing school at a single university in a single country. This limits generalisability, it may therefore be that the patterns and possible issues observed in this study are particular to these courses in this university. The second limitation is that that the University is situated in a particular geography, it is in Devon in the far South West of England and so is relatively geographically distant from sources of students, the data on source of students and their destination may well be influenced by this. Third, the analyses completed of differences (other than in WP3) were limited by the data that were available to us that had been routinely collected by the university. The data may therefore have limited generalisability to other countries but the theme we have identified may well be pointers to further evaluation in different territories. It was positive that these administrative data were of good quality with high completeness levels, but some data that we would have liked to examine in more detail such as ethnicity were not available to us. Finally the response rate to the survey can be seen as low, which may limit the results and conclusions drawn.

There are however also strengths to the approach we have taken. The focus enabled us to access and layer multiple datasets to allow a view of the whole process from application to enrolment, through attrition, to graduation and employment. As such the data may well have value in workforce planning terms. Second, this method provides a proof of concept that such data aggregation and triangulation is possible and presents a model for studies in other universities or groups of universities. Third, we were able to investigate elements of the effect of social deprivation by using postcode data, enabling initial investigation into the social inequalities in nursing training. Finally, the emerging data, while in many ways not definitive except in local terms, are useful in generating hypotheses to be tested in further research (e.g. on the effect of gender, age, and deprivation) and these data support the development of further evaluative research.

## Conclusions

The data provide useful information on the nursing educational pipeline. The findings that local students almost exclusively stay in the local region, shows the value for the system as a whole, including employers, in supporting local nursing education. The extent to which this applies in more densely populated and better-connected areas of the country warrants further investigation. The location and catchment area of students needs to be factored into workforce planning. The sequential higher likelihood of female students to be enrolled and to graduate compared with their male peers is of concern. Attrition rates has been found to differ between nursing programmes. This is partially compensated by transfers onto the programmes from other universities. It raises the possibility of inequity in the system and a bias towards female students. The data discussed here raise questions that need to be addressed locally and nationally by further empirical research. This research provides a proof of concept that useful workforce insights data can be generated by triangulating multiple sources of routinely collected data with specific audit of characteristics of particular interest.

## Data Availability

All datasets generated and analysed during the audit are not publically available due to them being the property of the University of Plymouth. Hence restrictions apply to the availability of these data.
